# The Usefulness of Intracoronary Imaging in Patients with ST-Segment Elevation Myocardial Infarction

**DOI:** 10.3390/jcm12185892

**Published:** 2023-09-11

**Authors:** Grigoris V. Karamasis, Charalampos Varlamos, Despoina-Rafailia Benetou, Andreas S. Kalogeropoulos, Thomas R. Keeble, Grigorios Tsigkas, Iosif Xenogiannis

**Affiliations:** 1 Cardiology Department, Attikon University Hospital, National and Kapodistrian University of Athens Medical School, Rimini 1, Chaidari, 124 62 Athens, Greece; 2 Department of Cardiology, Essex Cardiothoracic Centre, Basildon SS16 5NL, UK; 3 Department of Cardiology, Mitera General Hospital, 151 23 Athens, Greece; 4 Medical Technology Research Centre, Anglia Ruskin School of Medicine, Chelmsford CM1 1SQ, UK; 5 Department of Cardiology, University Hospital of Patras, 265 04 Patras, Greece

**Keywords:** intracoronary imaging, IVUS, OCT, ACS, STEMI

## Abstract

Intracoronary imaging (ICI) modalities, namely intravascular ultrasound (IVUS) and optical coherence tomography (OCT), have shown to be able to reduce major adverse cardiovascular events in patients undergoing percutaneous coronary intervention (PCI). Nevertheless, patients with ST-segment elevation myocardial infarction (STEMI) have been practically excluded from contemporary large randomized controlled trials. The available data are limited and derive mostly from observational studies. Nevertheless, contemporary studies are in favor of ICI utilization in patients who undergo primary PCI. Regarding technical aspects of PCI, ICI has been associated with the implantation of larger stent diameters, higher balloon inflations and lower residual in-stent stenosis post-PCI. OCT, although used significantly less often than IVUS, is a useful tool in the context of myocardial infarction without obstructive coronary artery disease since, due to its high spatial resolution, it can identify the underlying mechanism of STEMI, and, thus, guide therapy. Stent thrombosis (ST) is a rare, albeit a potential lethal, complication that is expressed clinically as STEMI in the vast majority of cases. Use of ICI is encouraged with current guidelines in order to discriminate the mechanism of ST among stent malapposition, underexpansion, uncovered stent struts, edge dissections, ruptured neoatherosclerotic lesions and coronary evaginations. Finally, ICI has been proposed as a tool to facilitate stent deferring during primary PCI based on culprit lesion characteristics.

## 1. Introduction

ST-segment elevation myocardial infarction (STEMI) is among the most dramatic manifestations of coronary artery disease (CAD), associated with increased short- and long-term mortality [1,2]. Effective and timely flow restoration with primary percutaneous coronary intervention (PCI) is of paramount importance in order to rescue the jeopardized myocardium and improve prognosis [1]. The presence of a large thrombus, commonly found in the clinical setting of STEMI, can lead to suboptimal stent deployment (i.e., stent undersizing and malapposition), a condition associated with the devastating complication of stent thrombosis (ST) [3,4].

Intracoronary imaging (ICI), namely intravascular ultrasound (IVUS) and optical coherence tomography (OCT), has multiple applications in PCI: (1) identification of the culprit lesion in cases of ambiguity, (2) clarification of the underlying mechanism of acute coronary syndrome (ACS) (i.e., atherosclerosis vs. dissection; plaque rupture vs. plaque erosion), (3) recognition of the composition of the atheromatous plaque and thus guidance of lesion preparation strategy, (4) determination of the appropriate size of balloons and stents that will be applied, (5) selection of the appropriate landing zone by guiding stent implantation away from segments with excessive plaque burden and (6) evaluation of PCI results and determination of the need for balloon post-dilation and further stent placement in cases of stent underexpansion, strut malapposition, tissue protrusion, edge dissection and a geographical miss [5,6,7,8].

A plethora of studies support that ICI guidance is superior to angiography-guided PCI with respect to patient clinical outcomes, with the benefit being more pronounced for complex lesions [9]. Large contemporary randomized controlled trials (RCTs) (i.e., RENOVATE-COMPLEX-PCI trial, ULTIMATE trial and IVUS-XPL) support that ICI-guided PCI reduces the rate of major adverse cardiovascular events (MACE) [10,11,12]. Nevertheless, STEMI patients undergoing primary PCI have been commonly excluded or under-represented in major trials investigating ICI.

In the real world, use of ICI in the context of ACS remains low. In the United States, the reported percentage is <10%, while in East Asian populations, the use of ICI reaches 26% [13,14,15,16,17]. IVUS is by far (>95%) the most used ICI modality in comparison to OCT [13,15]. Specifically, for STEMI patients, ICI application remains also relatively low, ranging from 4 to 31% [14,15,16,17,18]. Multiple factors could explain the low utilization of ICI in the context of STEMI: higher operation cost, presumed procedural delays, lack of expertise and finally, lack of randomized data supporting an advantage of ICI over angiography-guided PCI in this subgroup of patients.

In this narrative review, we aimed to present the main literature concerning the utilization of ICI in primary PCI for STEMI and its potential benefits compared with conventional primary PCI. Furthermore, we describe the role of ICI in specific STEMI scenarios, such as myocardial infarction with non-obstructive coronary arteries (MINOCA) and ST.

The novelty of this review consists of the fact that it presents and discusses data specifically for STEMI (and not generally on ACS) for both imaging modalities (IVUS and OCT), describes the role of ICI in specific STEMI clinical scenarios and includes the new recent literature (like the just published (August 2023) ILUMIEN IV and ESC guidelines on ACS management).

## 2. Rationale of ICI Use during Primary PCI

STEMI is usually the result of an acute thrombotic occlusion of an epicardial coronary artery. Primary PCI aims to succeed at fast reperfusion targeting the angiographically identified culprit lesion [1]. However, the culprit lesion may not be obvious or lumen-compromising, or could be located proximally or distally to the angiographically apparent target lesion [19,20]. Moreover, STEMI is characterized by increased thrombus burden, vessel vasoconstriction secondary to the extensive inflammatory response and vessel undersizing distal to the stenosis due to lower intracoronary pressure [21,22]. The latter pose an extra challenge for stent placement during primary PCI and increase the risk for incomplete lesion coverage, stent undersizing and stent underexpansion or malapposition, increasing the risk for future target vessel failure. It has been shown that drug eluting stents (DES) implanted for STEMI have a higher frequency of malapposition and uncovered struts at follow up [23] and that stents selected during primary PCI are usually smaller compared to the actual vessel size [24]. Even when optimization with angiographically guided post-dilation is applied, the rates of stent under-deployment remain high [25].

ICI could be used to optimize stent deployment during primary PCI. It has been shown that ICI optimization targets used in PCI for stable patients also apply in ACS. In the HORIZON-AMI IVUS substudy, a smaller IVUS minimum stent area was an independent predictor of angiographic restenosis after primary PCI in patients with STEMI, similar to patients with stable CAD [26]. In the CLI-OPCI ACS substudy, a composite of OCT-defined suboptimal stent implantation features and residual intrastent plaque/thrombus protrusion were associated with an adverse outcome [27].

Furthermore, ICI could be used to characterize the pathological substrate of the culprit lesion: a plaque rupture or erosion and calcified nodule [28]. Identification of these pathologies can influence clinical decisions (e.g., direct stenting in lipid-only plaque, calcium modification in calcified nodules, conservative treatment for plaque erosion, etc.) [15]. OCT, compared to IVUS, provides greater resolution and enhanced wall anatomy visualization and tissue characterization [7,8]. Thus, OCT is better in identifying the underlying cause of STEMI, differentiating plaque rupture from erosion, a fact that may influence decision making [29]. Furthermore, due to its high resolution, OCT can discriminate between the two types of thrombi in STEMI patients; a red thrombus, which has high backscatter and high attenuation, and a white thrombus, characterized by signal-rich low backscatter and low attenuation. However, the clinical impact of thrombus type recognition has yet to be determined [30].

The recently published 2023 ESC guidelines for the management of ACS support the use of ICI. Based on the guidelines, ICI should be considered to guide PCI of the culprit lesion. Importantly, this recommendation does not discriminate between STEMI and NSTEMI or IVUS and OCT. Furthermore, ICI gets a IIb recommendation (“may be considered”) in cases where there is ambiguity regarding the culprit lesion. For this indication, the guidelines indicate OCT as the preferred modality [31].

## 3. Impact of ICI Use during Primary PCI on Outcomes

Data regarding clinical outcomes in STEMI patients treated with ICI-guided PCI are scarce. Firstly, there are no major RCTs focused on ICI use specifically in STEMI. Secondly, STEMI patients are mostly excluded from contemporary ICI RCTs. For example, the two largest IVUS RCTs (i.e., IVUS-XPL and ULTIMATE—each one including more than 1400 patients randomized to IVUS vs. coronary angiography guidance in long lesions and all comers, respectively) excluded STEMI patients presenting less than 24 h from pain onset, hindering conclusions regarding IVUS in primary PCI [11,12]. Additionally, it should be noted that even in trials including STEMI patients, this group was profoundly under-represented; in the recently published large RENOVATE-COMPLEX-PCI RCT, where ICI-guided complex PCI with IVUS or OCT reduced cardiac death, target vessel myocardial infarction (MI) and revascularization, STEMI comprised only 2.4% of the cases (40 out of 1639) [10]. The same applies to smaller trials, where acute MI patients were mostly excluded, a pattern seemingly followed by most studies published during the last decade. As a consequence, evidence for ICI use during primary PCI stems mainly from observational studies and registries rather than RCTs. Finally, studies including ACS or MI mostly refer to unstable angina or non-ST elevation myocardial infarction (NSTEMI) rather than STEMI. The RENOVATE-COMPLEX-PCI RCT is a typical example: ACS accounted for half of the recruited cases (50.8%); however, 32.6% were unstable angina cases, 15.6% were NSTEMI and only 2.4% were STEMI, as already discussed [10].

## 4. Studies of ICI during Primary PCI for STEMI

Older reports of ICI (mainly IVUS) registries were not encouraging for its use in MI patients. In a prospective observational study of 905 patients who underwent primary PCI, IVUS guidance did not improve the rates of the primary composite endpoint of death, MI and target lesion revascularization (14.3% vs. 14.5%; *p* = 0.94) or the rates of definite and probable ST (2.1% vs. 2.1%; *p* = 0.99) at 1 year [32]. In the first report of the Korea Acute Myocardial Infarction Registry (KAMIR) published in 2011, IVUS did not appear to improve prognoses in an MI cohort [33]. Of note, the number of treated vessels and stents used, stent length and stent diameter were increased in the IVUS-guided group. Similar findings were reported in the CREDO-Kyoto AMI registry that included 3028 patients admitted with STEMI and was published in 2016 [18]. The use of IVUS was associated with a significantly higher diameter of implanted stents; however, at the 5-year follow up, despite a numerical reduction in MACE and target vessel revascularization in the IVUS group, the difference was not statistically significant following adjustment for confounders [18]. The previous studies recruited patients more than 15 years ago, so they hardly reflect contemporary clinical practice.

In contemporary studies, the use of ICI has been associated with improved cardiovascular outcomes. In the second publication of the KAMIR in 2019, a larger cohort of 11,731 patients was reported (47% STEMI), of which 19.9% had undergone IVUS and 2.4% OCT [34]. In the propensity-score-matched analysis, patient-oriented (5.9 vs. 7.7%, HR: 0.74, 95% CI: 0.60–0.92; *p* = 0.006) and device-oriented (5.0 vs. 6.8%, HR: 0.72, 95% CI: 0.57–0.90; *p* = 0.004) composite endpoints were found to be lower in the group guided with ICI. The difference was attributed to the reduction in all-cause mortality (4.4 vs. 7.0%; *p* < 0.001) and cardiac mortality (3.3 vs. 5.2%; *p* < 0.001) [34].

In the large COREA-AMI registry that recruited 9846 patients with AMI (54.7% with STEMI) who underwent PCI, IVUS utilization led to a reduction in MACE (cardiovascular death, MI and target lesion revascularization) (HR: 0.779, 95% CI: 0.689–0.880; *p* < 0.001) [17]—a finding that was maintained both within, as well as beyond the first year following index PCI. Of interest, left main and chronic renal failure patients seemed to gain the greatest benefit of IVUS use and STEMI patients were benefited more than NSTEMI patients. In addition, IVUS utilization was not related to a longer door-to-balloon time regarding primary PCI (73.5 ± 24.4 vs. 76.7 ± 26.1, for angiography- vs. IVUS-guided, respectively; *p* = 0.241) [17].

In another large multicenter prospective nationwide registry from Korea, which included 13,104 MI patients (50.4% STEMI) who had PCI with the implantation of second generation DES, IVUS use (21% of the study’s population) was associated with a lower risk of target lesion failure at 3 years (4.8% vs. 8%; *p* < 0.001), driven mainly by cardiac death and target vessel MI [16]. Of note, while IVUS was less likely to be applied in STEMI patients, its usage was equally beneficial for the NSTEMI and the STEMI group.

A Japanese multicenter prospective registry (J-MINUET) investigated the rate of use and the impact on prognoses of IVUS or OCT-guided PCI during urgent revascularization for MI (mainly STEMI) [34]. Angiography, IVUS and OCT-guided PCI were performed in 689 (24.7%), 1947 (69.8%) and 152 (5.5%) patients, respectively. In-hospital mortality was 10.4%, 5.1% and 3.3%, respectively (*p* < 0.01). In a univariate and multivariate logistic regression analysis, IVUS guidance (vs. angiography guidance, OR: 0.49, 95% CI: 0.30–0.81; *p* = 0.006) was independently associated with in-hospital mortality [35].

An observational study from the United States analyzed data from the Nationwide Readmissions Database (NRD) of STEMI patients who underwent PCI [36]. IVUS-guided PCI was applied in 33,644 (4.2%) of 809,601 STEMI cases. After 1:1 matching of patients with IVUS-guided PCI and patients with angiography-guided PCI, IVUS resulted in lower in-hospital mortality (3.9% vs. 4.6%; *p* < 0.0001) and lower rates of readmission due to acute MI at 6 and 11 months (5.7% vs. 6%, *p* = 0.045, and 5.1% vs. 6.5%, *p* = 0.005, respectively). Furthermore, PCI and mortality at 11 months (2.1% vs. 3%, *p* = 0.008, and 0.7% vs. 1.4%, *p* = 0.002, respectively) were lower in the IVUS-guided group [36].

In another observational study from the United States, Megaly et al. collected data from 252,970 STEMI patients using the National Inpatients Sample (NIS) database, in 5.5% of whom imaging was performed (96.4% IVUS) [15]. ICI usage was more frequent in patients with acute ST, anterior STEMI or patients likely to be diagnosed with spontaneous artery dissection. After propensity score matching, ICI use was related with a reduction in in-hospital mortality (3.6% vs. 4.8%; *p* = 0.010), increasing at the same time the cost of index hospitalization to USD 4703 more [15].

In a prospective substudy of the TOTAL RCT, 214 STEMI patients who received primary PCI guided with OCT were compared after 2:1 propensity matching with 428 patients who had PCI performed with angiography guidance alone [37]. The use of OCT resulted in a larger final in-stent minimum lumen diameter (2.99 ± 0.48 mm in the OCT-guided group versus 2.79 ± 0.47 mm in the angiography-guided group; *p* < 0.0001) while clinical outcomes at 1 year were similar between the two groups (7.5% of the OCT-guided group versus 9.8% of the angiography-guided group, HR: 0.76, 95% CI: 0.43–1.34; *p* = 0.34). However, the study did not have adequate power for examining clinical events [37].

Another RCT aspired to evaluate the possible benefits of OCT guidance in primary PCI [38]. Unfortunately, the study was prematurely terminated after recruiting 201 STEMI patients due to budget restriction, lacking power to assess clinical outcomes. Post-primary PCI optimization was performed in 29% of cases in the OCT group (59% malapposition and 41% dissections). An OCT analysis at 9 months showed that OCT use was associated with a lesser in-segment area of stenosis (6% [–11, 19] vs. 18% [3, 33]; *p* = 0.0002). No significant difference was found at 9 months regarding MACE rates (3% in the OCT group vs. 2% in the angio-guided group; *p* = 0.87) [38].

A recently published meta-analysis, dedicated to AMI patients, showed that IVUS-guided PCI significantly reduced the risk for all-cause mortality (pooled RR: 0.70) and MACE (pooled RR: 0.86) compared to angio-guided PCI [39]. The subset of patients with STEMI were also benefitted with IVUS guidance: all-cause mortality (pooled RR: 0.79, 95% CI: 0.66–0.95; *p* = 0.01) and MACE (pooled RR: 0.86, 95% CI: 0.74–0.99; *p* = 0.04) [39].

## 5. Future Studies

Large RCTs are warranted to prove the potential benefits of ICI-guided primary PCI for STEMI as suggested with the previously mentioned studies. Unfortunately, important RCTs that are currently running, assessing the use of OCT or IVUS-guided PCI in large patient cohorts (i.e., ILUMIEN IV—NCT03507777; IMPROVE—NCT04221815; and IVUS-CHIP—NCT04854070), essentially exclude STEMI patients undergoing primary PCI. In the well-anticipated ILUMINEN-IV RCT, PCI guided with OCT led to a larger minimum stent area compared to angiographically guided PCI [40]. However, there was no significant difference between the two strategies regarding the primary composite clinical endpoint of cardiac death, target-vessel MI or revascularization at 2 years. This came as a surprise, especially considering that the investigators aimed to recruit high-risk patients or patients with high-risk lesions. They attributed the neutral clinical result mainly to the unexpectedly low incidence of ischemia-driven target-vessel revascularization at the medium-term follow up (5.6% in each group). Nevertheless, OCT guidance resulted in less stent thrombosis (0.5% vs. 1.4%; *p* = 0.02) [40]. Patients with recent STEMI consisted of 5.7% of the study cohort. However, culprit lesion PCI for STEMI was included in the study only when it occurred more than 24 h after symptom onset. This detail essentially precludes patients who underwent primary PCI. In any case, the very small percentage of STEMI cases does not allow for drawing conclusions regarding the role of ICI in STEMI management.

Several ongoing trials will provide important data regarding ICI-guided primary PCI. The large iSTEMI (Intravascular Ultrasound Guided PCI in STEMI—NCT04775914) (*n* = 2500) investigates whether IVUS PCI will improve the clinical outcome of STEMI patients treated with primary PCI. OCT-CONTACT (OCT-guided vs. complete PCI in patients with ST-segment elevation myocardial infarction and multivessel disease—NCT04878133) is an RCT (*n* = 460) aiming to evaluate the effective benefit of OCT-guided vs. complete PCI in STEMI patients with multivessel coronary artery disease. SPECTRUM (Tissue Characterization and Primary Percutaneous Coronary Intervention Guidance Using Intravascular Ultrasound—NCT05007535) is an observational cohort study (*n* = 200) designed to assess the safety and efficacy of high-definition IVUS as guidance for primary PCI as well as culprit lesion plaque characteristics and thrombus morphology in patients with STEMI. The ATLAS-OCT trial is seeking to evaluate the feasibility of OCT guidance in STEMI patients undergoing PCI as a prospective, multicenter registry of consecutive STEMI patients who had primary PCI [41].

## 6. Special Clinical Scenarios

### 6.1. MINOCA

Advances in intravascular imaging and their liberal use in a catheterization laboratory have put MINOCA under the spotlight. MINOCA is not a benign condition as was previously thought, with 1-year mortality estimated at 3.6–4.7% [42,43,44]. According to a recent study from the United Kingdom dedicated to MINOCA patients with STEMI, all-cause mortality at 1 year was 4.5% [45]. The updated definition of MINOCA [46] requires the fulfilment of the following three criteria: (1) an acute MI diagnosis according to the “Fourth Universal Definition of Myocardial Infarction.” [47], (2) non-obstructive coronary arteries on coronary angiography defined as no lesions ≥50% in a major epicardial vessel and (3) the absence of another specific alternate diagnosis for the clinical presentation such as non-cardiac conditions (i.e., sepsis and pulmonary embolism) or non-ischemic causes (i.e., myocarditis, takotsubo syndrome and other cardiomyopathies).

**Figure 1:**
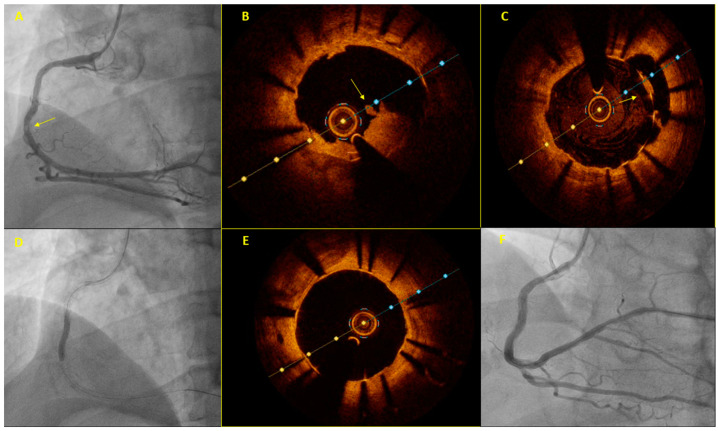
A sixty-year-old patient was referred for primary PCI due to inferior STEMI. He had a stent implanted in the right coronary artery (RCA) 11 years earlier. The initial coronary angiography showed no significant stenosis in the coronary arteries. A careful review of the angiogram revealed an area of “haziness” in the mid-RCA (arrow, **A**). OCT illustrated the presence of a non-obstructive red thrombus in the corresponding RCA segment (arrow, **B**) within the previously implanted stent, which appeared to be underexpanded and malapposed (arrow, **C**). Dilation with a non-compliant 3.5 mm balloon and a drug-coated 3.5 mm Agent balloon followed (**D**). Repeat OCT showed a significantly better stent expansion and apposition (**E**) with an excellent final angiographic result (**F**).

It is worth noting that MINOCA should be considered by the treating physician as a working diagnosis, rather than a final diagnosis, prompting further investigation, since under this term falls a group of diverse clinical entities with heterogenous pathogenetic mechanisms requiring individualized management [46,48]. Underlying causes can be divided into atherosclerotic (plaque rupture, plaque erosion and calcified nodule) and non-atherosclerotic (epicardial coronary vasospasm, spontaneous coronary artery dissection (SCAD), coronary embolism/thrombosis and microvascular dysfunction) [46]. Although current guidelines do not provide any specific recommendations with respect to ICI utilization in MINOCA cases, expert consensus documents favor its use (preferably OCT) since the identification of the underlying pathogenetic mechanism has a significant implication in short- and long-term patient management [8,46,49]. Figure 1 presents a characteristic example of ICI use in this cohort. In non-atherosclerotic MINOCA, ICI can reveal an intimal tear, false lumen or intramural hematoma in case of SCAD; intimal “bumping” or co-existing plaque erosion at the site of the coronary vasospasm; or an intact vessel wall in cases of a thromboembolism [8,46,49]. These findings in conjunction with the clinical scenario, the angiographic findings and the results of other imaging modalities could confirm or at least suggest the underlying diagnosis [46]. Medical therapy without stenting is the mainstay of treatment in the presence of non-atherosclerotic causes such as SCAD or a spasm. Recommendations for stent implantation in the clinical setting of MINOCA of an atherosclerotic cause are contradictory; a scientific statement from the American Heart Association on the contemporary diagnosis and management of patients with MINOCA argues against routine stent implantation for plaque disruption without differentiation between plaque rupture and plaque erosion [46]. On the contrary, an expert consensus document from the European Association of Percutaneous Cardiovascular Interventions favors stent implantation in the presence of a plaque rupture while it suggests withholding stenting in cases of plaque erosion without obstruction where flow has been restored [8].

The prevalence of MINOCA among patients with STEMI is 2.6–4.4% [19,45,50] and is higher among younger patients, females and those with fewer cardiovascular risk factors [19,51]. The rates of MINOCA in the COVID-19 era have increased; according to a report derived from the North American COVID-19 STEMI (NACMI) registry, STEMI without an identifiable culprit vessel reached 21% [52].

Older studies have used IVUS for the clarification of the underlying pathophysiologic mechanism of MINOCA. In a study conducted by Reynolds et al., plaque disruption was found in 2/6 (33%) patients presenting with STEMI [53]. Due to its superior spatial resolution compared with IVUS, OCT is the preferred imaging modality as it can visualize, in detail, intraluminal and coronary vessel wall microstructures and thus identify the underlying pathology [8]. Multiple studies have used OCT to delineate the pathogenetic mechanism of MINOCA [54,55,56,57]. However, none of these studies exclusively evaluated patients with STEMI but a “mixed” population suffering from ACS. Opolski et al. examined 38 patients with MINOCA, of whom 15 (39%) had STEMI [55]. In five (33%) STEMI patients, OCT managed to image disrupted plaque: three patients had ruptured plaque with a superimposed thrombus, one patient had ruptured plaque without a thrombus and one patient had a calcified nodule. Additionally, a thrombus without underlying disrupted plaque was found in the infarct-related artery of two patients. The left anterior descending coronary artery was the culprit artery in 6 out of 7 previously mentioned cases. Tarya et al. recruited 82 ACS patients without obstructive CAD [57]. Ten (12%) patients were diagnosed with STEMI and in seven of these patients, OCT revealed a hidden high-risk lesion, defined as ruptured plaque, plaque erosion, a calcified nodule, SCAD, a lone thrombus or thin-cap fibroatheroma. Finally, in the largest conducted study in the field, Reynolds et al. examined the underlying cause of MINOCA in 170 women with the use of multivessel OCT followed by cardiac magnetic resonance [56]. Interestingly, only 5 patients with STEMI were included in the study with OCT identifying a culprit lesion (plaque rupture, plaque erosion, intraplaque cavity, layered plaque, SCAD or intimal bumping) in 2 (40%) of them. Patients with STEMI in the previous studies were too few to allow definite conclusions about the prevalence and the type of culprit lesions in patients with STEMI and non-obstructive CAD, highlighting the need for larger studies dedicated to this subgroup of MINOCA patients. In addition, differences in methodology (single vs. multivessel OCT) and definition of unstable plaque may have, at least partially, accounted for the diverse findings.

### 6.2. Stent Thrombosis

In the era of the modern stent platforms and potent antiplatelets, ST is a rare, albeit potentially deadly, complication. The early and late ST percentage is estimated to be <1% [58,59]. Second generation DES have overcome the first generation DES Achilles’ hill, namely very late ST, with its incidence, according to a recent large meta-analysis, being 0.9% [60]. ST has an adverse prognosis with up to 45% mortality [61]. The majority of patients (up to 80–90%) present with STEMI that is associated with higher mortality compared with de novo STEMI [62,63].

**Figure 2:**
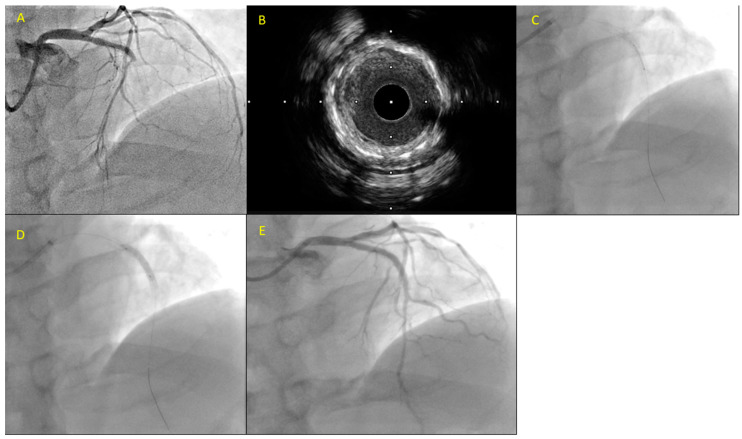
A fifty-three-year-old patient presented with substernal chest pain and ST-segment elevation in leads V2 through V6. He had a history significant for anterior STEMI with a DES placement in the LAD 3 years prior. The patient was led to the catheterization laboratory where he was diagnosed with in-stent thrombosis (**A**). After predilations with a 2.5 mm non-compliant balloon, intravascular ultrasound (IVUS) was performed showing stent underexpansion and neoatherosclerosis. (**B**) A new DES, a 3.0 mm × 12 mm DES, was delivered inside the previously implanted stent (**C**) followed by postdilations with a 3.5 mm non-compliant balloon (**D**) with a good final result (**E**).

The European and American guidelines for coronary artery revascularization recommend the use of IVUS or OCT in case of stent failure for the determination of an ST mechanism [64,65]. Figure 2 presents an example of ST treated with ICI-guided primary PCI. Recent large ICI imaging studies have shed light on stent deployment factors related to ST. Depending on the revealed mechanism of ST (i.e., stent underexpansion or malapposition vs. edge dissection, a geographical miss or neoatherosclerosis), different management can be applied—aggressive, high-pressure balloon dilation for the former or new stent placement for the latter mechanism [62,66,67]. Due to its higher spatial resolution, OCT can distinguish a thrombus from other tissue components and delineate stent structure and deployment features better than IVUS; thus, it is considered the preferred imaging modality for ST [7]. Nevertheless, a bulky thrombus can lead to light attenuation obscuring the visualization of stent struts and the outer vessel wall with OCT [7]. Restoration of TIMI flow III with thrombectomy and/or GPIIb-IIIa inhibitor administration can enhance the chances of discrimination of the underlying pathogenetic ST mechanism with OCT [7,68].

Lee et al. compared the IVUS findings of a very late stent thrombosis in 30 patients after either DES (*n* = 23) or BMS (*n* = 7) implantation [69]. Most patients (80%) presented with STEMI. Although minimal stent cross-sectional area (CSA) was similar between the two groups, mean stent CSA and mean neointimal CSA were smaller in the DES group than in the BMS group. Interestingly, stent malapposition was found in 17 DES patients (74%) and in no BMS patients (0%) while a neointimal rupture within the stent was noticed in 10 DES patients (43.5%) and 7 BMS patients (100%), leading the investigators to conclude that different pathogenetic mechanisms play a crucial role in the development of very late ST according to stent type. It should be noted that the previously mentioned study included only first generation DES.

Contemporary studies have utilized OCT to evaluate the pathophysiologic mechanisms of ST. The national PESTO (Morphological Parameters Explaining Stent Thrombosis assessed by OCT) French registry was designed to examine the characteristics and mechanisms of ST with the use of OCT [68]. In a recent report derived from the previously mentioned registry, OCT managed to delineate the underlying mechanism in 97% of 120 patients with ST. Most patients (82%) presented with STEMI. Concerning acute and subacute ST, malapposition was the predominant mechanism (48%) followed by severe underexpansion (26%), edge dissection (4%) and edge-related disease progression (4%). Stent malapposition was again the commonest ST finding in late and very late ST (32%), followed by ruptured neoatherosclerotic lesions (28%), coronary evaginations (10%) and isolated uncovered struts (10%). With respect to stent type, ruptured neoatherosclerotic lesions were more frequent with BMS compared with DES (36% vs. 14%; *p* = 0.005), while coronary evaginations were found more frequently in the DES than in the BMS group (13% vs. 3%; *p* = 0.04). Tanawaki et al. examined with OCT 58 patients with very late ST who had a previously implanted DES (66% had an early generation DES (sirolimus- and paclitaxel-eluting stents) and 33% had a newer generation DES (zotarolimus-, everolimus- and biolimus-eluting stents)) [70]. The most common clinical presentation was STEMI (78%). An underlying pathophysiologic mechanism was identified in 98% of patients. In accordance with the previously mentioned study, strut malapposition (34.5%) and neoatherosclerosis (27.6%) were the most frequently found causative mechanisms, followed by uncovered struts (12.1%) and stent underexpansion (6.9%). More than one mechanism of very late ST in the same lesion was observed in 55% of the cases. Finally, it is worth noting that the correlation of malapposition and uncovered stent struts with very late ST was consistent in early and newer generation DES, as was the frequency of neoatherosclerosis. The largest study on the topic to date, derived from the PRESTIGE Consortium (Prevention of Late Stent Thrombosis by an Interdisciplinary Global European Effort), included 62 patients with early and 155 patients with late or very late ST [71]. STEMI was the clinical presentation in 79% of the study’s subjects. A newer generation DES was the underlying stent type in approximately half of the patients. Regarding the causative mechanism according to time of ST presentation, uncovered (67%) and malapposed struts (27%) were the predominant mechanisms in acute ST while uncovered struts (62%) and stent underexpansion (26%) were the dominant causes in subacute ST. With respect to late and very late ST, uncovered struts (33%) and severe restenosis (19%) for the former and neoatherosclerosis (31%) and uncovered struts (20%) for the latter were the most common findings. In patients with very late ST, uncovered stent struts were the dominant mechanism for DES whereas neoatherosclerosis was the main mechanism for BMS.

### 6.3. Non-Stenting Strategy during Primary PCI Based on ICI (OCT) Findings

ICI utilization, particularly OCT, allows the operator to accurately assess the morphology of the culprit lesion in STEMI patients who undergo primary PCI. OCT can discriminate between plaque rupture or erosion, a calcified nodule and SCAD [7,8]. Some investigators argue that a conservative strategy without stenting could be an option when there is no significant residual stenosis after restoration of flow in specific substrates (like plaque erosion). Avoiding stent deployment could protect, for example, young patients from future target vessel or lesion failure [72]. This notion has been tested in the EROSION series of studies.

The pilot EROSION study included 55 patients with ACS due to plaque erosion with residual diameter stenosis <70% on coronary angiography after manual thrombectomy in 46 (83.6%) patients who were treated with anti-thrombotic therapy without stenting (GPIIb/IIIa inhibitor in 35 (63.6%) plus aspirin and ticagrelor) [73,74]. Most patients (96%) had STEMI. The vast majority of the patients (92.5%) experienced no MACE at 12 months. At a median follow up of 4.8 years, among 52 patients, there were no cases of death, MI, stroke, coronary artery bypass grafting or heart failure. However, 11 (21.1%) patients had elective target lesion revascularization [75]. A follow-up randomized controlled study, EROSION III, compared OCT vs. angiographic guidance with respect to the optimization of the reperfusion strategy in STEMI with angiographic diameter stenosis ≤ 70% and TIMI flow grade III [72]. A non-stenting strategy was suggested for plaque erosions, certain ruptures with no obvious dissection and/or hematoma and SCAD. Among 112 patients in the OCT group, a plaque rupture was the predominant type of unstable plaque found in 74 (66.1%) patients followed by plaque erosion in 29 (25.9%) and a calcified nodule in 5 (4.5%) subjects. One (0.9%) patient was diagnosed with an intimal fissure while in 3 (2.7%) patients, there was the absence of an atherosclerotic lesion or residual thrombus. OCT guidance led to a 15% decrease in stent implantation during primary PCI with no increase in major cardiocerebrovascular events at the 1-year follow up. Specifically, stent implantation took place in 59% of plaque rupture cases and in only 14% and 20% of plaque erosion and calcified nodule cases, respectively.

EROSION studies showed that, apart from stent optimization, OCT has a role in guiding treatment according to the morphologic characteristics of the culprit lesion. Plaque erosion has been related with less favorable stent healing compared with a plaque rupture [76]. The presence of severe calcification, as in calcified nodules, hinders appropriate stent expansion and apposition, resulting in worse long-term outcomes [77,78]; thus, if stent placement is deemed to be necessary, aggressive lesion preparation with cutting balloons, atherectomy, a laser or lithotripsy should precede stenting [8]. Taking under consideration the previous, deferring stenting in cases of plaque erosion and calcified nodules could be considered to protect patients from subsequent stent-related complications.

## 7. Conclusions

In conclusion, the use of ICI in the context of STEMI and primary PCI appears to have a potential benefit on clinical outcomes. However, the data are limited and derived mostly from observational studies. Thus, STEMI-dedicated, large-scale RCTs are needed to elaborate the optimal use of ICI in these high-risk patients. Nevertheless, ICI can be extremely useful in specific subsets of STEMI cases, such as MINOCA and ST, where it can delineate the underlying pathophysiologic mechanism and guide treatment.

## References

[1] Ibanez B., James S., Agewall S., Antunes M.J., Bucciarelli-Ducci C., Bueno H., Caforio A.L.P., Crea F., Goudevenos J.A., Halvorsen S. (2018). 2017 ESC Guidelines for the management of acute myocardial infarction in patients presenting with ST-segment elevation: The Task Force for the management of acute myocardial infarction in patients presenting with ST-segment elevation of the European Society of Cardiology (ESC). Eur. Heart J..

[2] Klancik V., Pesl L., Neuberg M., Tousek P., Kocka V. (2022). Long-term follow-up in patients with ST-segment elevation myocardial infarction who underwent primary percutaneous coronary intervention. Eur. Heart J. Suppl..

[3] Généreux P., Stone G.W., Harrington R.A., Gibson C.M., Steg P.G., Brener S.J., Angiolillo D.J., Price M.J., Prats J., LaSalle L. (2014). Impact of intraprocedural stent thrombosis during percutaneous coronary intervention: Insights from the CHAMPION PHOENIX Trial (Clinical Trial Comparing Cangrelor to Clopidogrel Standard of Care Therapy in Subjects Who Require Percutaneous Coronary Intervention). J. Am. Coll. Cardiol..

[4] Brilakis E.S. (2020). Manual of Percutaneous Coronary Interventions: A Step-by-Step Approach.

[5] Mintz G.S., Matsumura M., Ali Z., Maehara A. (2022). Clinical Utility of Intravascular Imaging: Past, Present, and Future. JACC Cardiovasc. Imaging.

[6] Truesdell A.G., Alasnag M.A., Kaul P., Rab S.T., Riley R.F., Young M.N., Batchelor W.B., Maehara A., Welt F.G., Kirtane A.J. (2023). Intravascular Imaging During Percutaneous Coronary Intervention: JACC State-of-the-Art Review. J. Am. Coll. Cardiol..

[7] Räber L., Mintz G.S., Koskinas K.C., Johnson T.W., Holm N.R., Onuma Y., Radu M.D., Joner M., Yu B., Jia H. (2018). Clinical use of intracoronary imaging. Part 1: Guidance and optimization of coronary interventions. An expert consensus document of the European Association of Percutaneous Cardiovascular Interventions. Eur. Hear. J..

[8] Johnson T.W., Räber L., Di Mario C., Bourantas C., Jia H., Mattesini A., Gonzalo N., Hernandez J.M.D.L.T., Prati F., Koskinas K. (2019). Clinical use of intracoronary imaging. Part 2: Acute coronary syndromes, ambiguous coronary angiography findings, and guiding interventional decision-making: An expert consensus document of the European Association of Percutaneous Cardiovascular Interventions. Eur. Heart J..

[9] Mintz G.S., Bourantas C.V., Chamié D. (2022). Intravascular Imaging for Percutaneous Coronary Intervention Guidance and Optimization: The Evidence for Improved Patient Outcomes. J. Soc. Cardiovasc. Angiogr. Interv..

[10] Lee J.M., Choi K.H., Bin Song Y., Lee J.-Y., Lee S.-J., Lee S.Y., Kim S.M., Yun K.H., Cho J.Y., Kim C.J. (2023). Intravascular Imaging–Guided or Angiography-Guided Complex PCI. N. Engl. J. Med..

[11] Gao X.-F., Ge Z., Kong X.-Q., Kan J., Han L., Lu S., Tian N.-L., Lin S., Lu Q.-H., Wang X.-Y. (2021). 3-Year Outcomes of the ULTIMATE Trial Comparing Intravascular Ultrasound Versus Angiography-Guided Drug-Eluting Stent Implantation. JACC Cardiovasc. Interv..

[12] Hong S.-J., Mintz G.S., Ahn C.-M., Kim J.-S., Kim B.-K., Ko Y.-G., Kang T.-S., Kang W.-C., Kim Y.H., Hur S.-H. (2020). Effect of Intravascular Ultrasound-Guided Drug-Eluting Stent Implantation: 5-Year Follow-Up of the IVUS-XPL Randomized Trial. JACC Cardiovasc. Interv..

[13] Smilowitz N.R., Mohananey D., Razzouk L., Weisz G., Slater J.N. (2018). Impact and trends of intravascular imaging in diagnostic coronary angiography and percutaneous coronary intervention in inpatients in the United States. Catheter. Cardiovasc. Interv..

[14] Mentias A., Sarrazin M.V., Saad M., Panaich S., Kapadia S., Horwitz P.A., Girotra S. (2020). Long-Term Outcomes of Coronary Stenting with and without Use of Intravascular Ultrasound. JACC: Cardiovasc. Interv..

[15] Megaly M., Pershad A., Glogoza M., Elbadawi A., Omer M., Saad M., Mentias A., Elgendy I., Burke M.N., Capodanno D. (2020). Use of Intravascular Imaging in Patients With ST-Segment Elevation Acute Myocardial Infarction. Cardiovasc. Revasc. Med..

[16] Kim Y., Bae S., Johnson T.W., Son N., Sim D.S., Hong Y.J., Kim S.W., Cho D., Kim J., Kim B. (2022). Role of Intravascular Ultrasound-Guided Percutaneous Coronary Intervention in Optimizing Outcomes in Acute Myocardial Infarction. J. Am. Heart Assoc..

[17] Choi I.J., Lim S., Choo E.H., Hwang B.-H., Kim C.J., Park M.-W., Lee J.-M., Park C.S., Kim H.Y., Yoo K.-D. (2021). Impact of Intravascular Ultrasound on Long-Term Clinical Outcomes in Patients with Acute Myocardial Infarction. JACC Cardiovasc. Interv..

[18] Nakatsuma K., Shiomi H., Morimoto T., Ando K., Kadota K., Watanabe H., Taniguchi T., Yamamoto T., Furukawa Y., Nakagawa Y. (2016). Intravascular Ultrasound Guidance vs. Angiographic Guidance in Primary Percutaneous Coronary Intervention for ST-Segment Elevation Myocardial Infarction—Long-Term Clinical Outcomes From the CREDO-Kyoto AMI Registry. Circ. J..

[19] Andersson H.B., Pedersen F., Engstrøm T., Helqvist S., Jensen M.K., Jørgensen E., Kelbæk H., Räder S.B.E.W., Saunamäki K., Bates E. (2017). Long-term survival and causes of death in patients with ST-elevation acute coronary syndrome without obstructive coronary artery disease. Eur. Heart J..

[20] Cheneau E., Leborgne L., Mintz G.S., Kotani J.-I., Pichard A.D., Satler L.F., Canos D., Castagna M., Weissman N.J., Waksman R. (2003). Predictors of subacute stent thrombosis: Results of a systematic intravascular ultrasound study. Circulation.

[21] Virmani R., Kolodgie F.D., Burke A.P., Farb A., Schwartz S.M. (2000). Lessons from Sudden Coronary Death: A Comprehensive Morphological Classification Scheme for Atherosclerotic Lesions. Arterioscler. Thromb. Vasc. Biol..

[22] Muller O., Pyxaras S.A., Trana C., Mangiacapra F., Barbato E., Wijns W., Taylor C.A., De Bruyne B. (2012). Pressure–Diameter Relationship in Human Coronary Arteries. Circ. Cardiovasc. Interv..

[23] Gonzalo N., Barlis P., Serruys P.W., Garcia-Garcia H.M., Onuma Y., Ligthart J., Regar E. (2009). Incomplete Stent Apposition and Delayed Tissue Coverage Are More Frequent in Drug-Eluting Stents Implanted During Primary Percutaneous Coronary Intervention for ST-Segment Elevation Myocardial Infarction Than in Drug-Eluting Stents Implanted for Stable/Unstable Angina: Insights from Optical Coherence Tomography. JACC Cardiovasc. Interv..

[24] Carrick D., Oldroyd K.G., McEntegart M., Haig C., Petrie M.C., Eteiba H., Hood S., Owens C., Watkins S., Layland J. (2014). A Randomized Trial of Deferred Stenting Versus Immediate Stenting to Prevent No- or Slow-Reflow in Acute ST-Segment Elevation Myocardial Infarction (DEFER-STEMI). J. Am. Coll. Cardiol..

[25] Karamasis G.V., Kalogeropoulos A.S., Gamma R.A., Clesham G.J., Marco V., Tang K.H., Jagathesan R., Sayer J.W., Robinson N.M., Kabir A. (2021). Effects of stent postdilatation during primary PCI for STEMI: Insights from coronary physiology and optical coherence tomography. Catheter. Cardiovasc. Interv..

[26] Choi S.-Y., Maehara A., Cristea E., Witzenbichler B., Guagliumi G., Brodie B., Kellett M.A., Dressler O., Lansky A.J., Parise H. (2012). Usefulness of Minimum Stent Cross Sectional Area as a Predictor of Angiographic Restenosis After Primary Percutaneous Coronary Intervention in Acute Myocardial Infarction (from the HORIZONS-AMI Trial IVUS Substudy). Am. J. Cardiol..

[27] Prati F., Romagnoli E., Gatto L., La Manna A., Burzotta F., Limbruno U., Versaci F., Fabbiocchi F., Di Giorgio A., Marco V. (2016). Clinical Impact of Suboptimal Stenting and Residual Intrastent Plaque/Thrombus Protrusion in Patients with Acute Coronary Syndrome: The CLI-OPCI ACS Substudy (Centro per la Lotta Contro L’Infarto-Optimization of Percutaneous Coronary Intervention in Acute Coronary Syndrome). Circ. Cardiovasc. Interv..

[28] Kajander O.A., Pinilla-Echeverri N., Jolly S.S., Bhindi R., Huhtala H., Niemelä K., Fung A., Vijayaraghavan R., Alexopoulos D., Sheth T. (2016). Culprit plaque morphology in STEMI—An optical coherence tomography study: Insights from the TOTAL-OCT substudy. EuroIntervention.

[29] Prati F., Uemura S., Souteyrand G., Virmani R., Motreff P., Di Vito L., Biondi-Zoccai G., Halperin J., Fuster V., Ozaki Y. (2013). OCT-Based Diagnosis and Management of STEMI Associated with Intact Fibrous Cap. JACC Cardiovasc. Imaging.

[30] Kume T., Akasaka T., Kawamoto T., Ogasawara Y., Watanabe N., Toyota E., Neishi Y., Sukmawan R., Sadahira Y., Yoshida K. (2006). Assessment of Coronary Arterial Thrombus by Optical Coherence Tomography. Am. J. Cardiol..

[31] A Byrne R., Rossello X., Coughlan J.J., Barbato E., Berry C., Chieffo A., Claeys M.J., Dan G.-A., Dweck M.R., Galbraith M. (2023). 2023 ESC Guidelines for the management of acute coronary syndromes. Eur. Heart J..

[32] Maluenda G., Lemesle G., Ben-Dor I., Collins S.D., Syed A.I., Torguson R., Kaneshige K., Xue Z., Suddath W.O., Satler L.F. (2010). Impact of intravascular ultrasound guidance in patients with acute myocardial infarction undergoing percutaneous coronary intervention. Catheter. Cardiovasc. Interv..

[33] Ahmed K., Jeong M.H., Chakraborty R., Ahn Y., Sim D.S., Park K., Hong Y.J., Kim J.H., Cho K.H., Kim M.C. (2011). Role of Intravascular Ultrasound in Patients with Acute Myocardial Infarction Undergoing Percutaneous Coronary Intervention. Am. J. Cardiol..

[34] Kim N., Lee J.H., Jang S.Y., Bae M.H., Yang D.H., Park H.S., Cho Y., Jeong M.H., Park J., Kim H. (2020). Intravascular modality-guided versus angiography-guided percutaneous coronary intervention in acute myocardial infarction. Catheter. Cardiovasc. Interv..

[35] Okura H., Saito Y., Soeda T., Nakao K., Ozaki Y., Kimura K., Ako J., Noguchi T., Yasuda S., Suwa S. (2019). Frequency and prognostic impact of intravascular imaging-guided urgent percutaneous coronary intervention in patients with acute myocardial infarction: Results from J-MINUET. Heart Vessel..

[36] Ya’Qoub L., Gad M., Saad A.M., Elgendy I.Y., Mahmoud A.N. (2021). National trends of utilization and readmission rates with intravascular ultrasound use for ST-elevation myocardial infarction. Catheter. Cardiovasc. Interv..

[37] Sheth T.N., Kajander O.A., Lavi S., Bhindi R., Cantor W.J., Cheema A.N., Stankovic G., Niemelä K., Natarajan M.K., Shestakovska O. (2016). Optical Coherence Tomography-Guided Percutaneous Coronary Intervention in ST-Segment-Elevation Myocardial Infarction: A Prospective Propensity-Matched Cohort of the Thrombectomy Versus Percutaneous Coronary Intervention Alone Trial. Circ. Cardiovasc. Interv..

[38] Kala P., Cervinka P., Jakl M., Kanovsky J., Kupec A., Spacek R., Kvasnak M., Poloczek M., Cervinkova M., Bezerra H. (2018). OCT guidance during stent implantation in primary PCI: A randomized multicenter study with nine months of optical coherence tomography follow-up. Int. J. Cardiol..

[39] Groenland F.T., Neleman T., Kakar H., Scoccia A., Plantes A.C.Z.D., Clephas P.R., Chatterjee S., Zhu M., Dekker W.K.D., Diletti R. (2022). Intravascular ultrasound-guided versus coronary angiography-guided percutaneous coronary intervention in patients with acute myocardial infarction: A systematic review and meta-analysis. Int. J. Cardiol..

[40] Ali Z.A., Landmesser U., Maehara A., Matsumura M., Shlofmitz R.A., Guagliumi G., Price M.J., Hill J.M., Akasaka T., Prati F. (2023). Optical Coherence Tomography–Guided versus Angiography-Guided PCI. N. Engl. J. Med..

[41] Yonetsu T., Wakabayashi K., Mizukami T., Yamamoto M.H., Yasuhara S., Kondo S., Oishi Y., Okabe T., Sugiyama T., Araki M. (2023). Optical Coherence Tomography–Guided Percutaneous Coronary Intervention for ST-Segment Elevation Myocardial Infarction: Rationale and Design of the ATLAS-OCT Study. Am. J. Cardiol..

[42] Bainey K.R., Welsh R.C., Alemayehu W., Westerhout C.M., Traboulsi D., Anderson T., Brass N., Armstrong P.W., Kaul P. (2018). Population-level incidence and outcomes of myocardial infarction with non-obstructive coronary arteries (MINOCA): Insights from the Alberta contemporary acute coronary syndrome patients invasive treatment strategies (COAPT) study. Int. J. Cardiol..

[43] Hjort M., Lindahl B., Baron T., Jernberg T., Tornvall P., Eggers K.M. (2018). Prognosis in relation to high-sensitivity cardiac troponin T levels in patients with myocardial infarction and non-obstructive coronary arteries. Am. Heart J..

[44] Pasupathy S., Air T., Dreyer R.P., Tavella R., Beltrame J. (2015). F Systematic review of patients presenting with suspected myocardial infarction and nonobstructive coronary arteries. Circulation.

[45] Gue Y.X., Corballis N., Ryding A., Kaski J.C., Gorog D.A. (2019). MINOCA presenting with STEMI: Incidence, aetiology and outcome in a contemporaneous cohort. J. Thromb. Thrombolysis.

[46] Tamis-Holland J.E., Jneid H., Reynolds H.R., Agewall S., Brilakis E.S., Brown T.M., Lerman A., Cushman M., Kumbhani D.J., Arslanian-Engoren C. (2019). Contemporary Diagnosis and Management of Patients with Myocardial Infarction in the Absence of Obstructive Coronary Artery Disease: A Scientific Statement from the American Heart Association. Circulation.

[47] Thygesen K., Alpert J.S., Jaffe A.S., Chaitman B.R., Bax J.J., Morrow D.A., White H.D. (2018). Fourth Universal Definition of Myocardial Infarction (2018). Glob. Heart.

[48] Collet J.-P., Thiele H., Barbato E., Barthélémy O., Bauersachs J., Bhatt D.L., Dendale P., Dorobantu M., Edvardsen T., Folliguet T. (2021). 2020 ESC Guidelines for the management of acute coronary syndromes in patients presenting without persistent ST-segment elevation. Eur. Heart J..

[49] Karamasis G., Xenogiannis I., Varlamos C., Deftereos S., Alexopoulos D. (2022). Use of Optical Coherence Tomography in MI with Non-obstructive Coronary Arteries. Interv. Cardiol. Rev. Res. Resour..

[50] Widimsky P., Stellova B., Groch L., Aschermann M., Branny M., Zelizko M., Stasek J., Formanek P. (2006). Prevalence of normal coronary angiography in the acute phase of suspected ST-elevation myocardial infarction: Experience from the PRAGUE studies. Can. J. Cardiol..

[51] Rallidis L.S., Xenogiannis I., Brilakis E.S., Bhatt D.L. (2022). Causes, Angiographic Characteristics, and Management of Premature Myocardial Infarction: JACC State-of-the-Art Review. J. Am. Coll. Cardiol..

[52] Quesada O., Van Hon L., Yildiz M., Madan M., Sanina C., Davidson L., Htun W.W., Saw J., Garcia S., Dehghani P. (2022). Sex Differences in Clinical Characteristics, Management Strategies, and Outcomes of STEMI With COVID-19: NACMI Registry. J. Soc. Cardiovasc. Angiogr. Interv..

[53] Reynolds H.R., Srichai M.B., Iqbal S.N., Slater J.N., Mancini G.J., Feit F., Pena-Sing I., Axel L., Attubato M.J., Yatskar L. (2011). Mechanisms of Myocardial Infarction in Women Without Angiographically Obstructive Coronary Artery Disease. Circulation.

[54] Gerbaud E., Arabucki F., Nivet H., Barbey C., Cetran L., Chassaing S., Seguy B., Lesimple A., Cochet H., Montaudon M. (2020). OCT and CMR for the Diagnosis of Patients Presenting with MINOCA and Suspected Epicardial Causes. JACC Cardiovasc. Imaging.

[55] Opolski M.P., Spiewak M., Marczak M., Debski A., Knaapen P., Schumacher S.P., Staruch A.D., Grodecki K., Chmielak Z., Lazarczyk H. (2019). Mechanisms of Myocardial Infarction in Patients with Nonobstructive Coronary Artery Disease: Results from the Optical Coherence Tomography Study. JACC Cardiovasc. Imaging.

[56] Reynolds H.R., Maehara A., Kwong R.Y., Sedlak T., Saw J., Smilowitz N.R., Mahmud E., Wei J., Marzo K., Matsumura M. (2021). Coronary Optical Coherence Tomography and Cardiac Magnetic Resonance Imaging to Determine Underlying Causes of Myocardial Infarction with Nonobstructive Coronary Arteries in Women. Circulation.

[57] Taruya A., Tanaka A., Nishiguchi T., Ozaki Y., Kashiwagi M., Yamano T., Matsuo Y., Ino Y., Kitabata H., Takemoto K. (2020). Lesion characteristics and prognosis of acute coronary syndrome without angiographically significant coronary artery stenosis. Eur. Heart J. Cardiovasc. Imaging.

[58] Kang S.-H., Chae I.-H., Park J.-J., Lee H.S., Kang D.-Y., Hwang S.-S., Youn T.-J., Kim H.-S. (2016). Stent Thrombosis with Drug-Eluting Stents and Bioresorbable Scaffolds: Evidence from a Network Meta-Analysis of 147 Trials. JACC Cardiovasc. Interv..

[59] Batchelor R., Dinh D., Brennan A., Lefkovits J., Reid C., Duffy S.J., Cox N., Liew D., Stub D. (2020). Incidence, Predictors and Clinical Outcomes of Stent Thrombosis Following Percutaneous Coronary Intervention in Contemporary Practice. Heart Lung Circ..

[60] Madhavan M.V., Kirtane A.J., Redfors B., Généreux P., Ben-Yehuda O., Palmerini T., Benedetto U., Biondi-Zoccai G., Smits P.C., von Birgelen C. (2020). Stent-Related Adverse Events >1 Year After Percutaneous Coronary Intervention. J. Am. Coll. Cardiol..

[61] Byrne R.A., Joner M., Kastrati A. (2015). Stent thrombosis and restenosis: What have we learned and where are we going? The Andreas Grüntzig Lecture ESC 2014. Eur. Heart J..

[62] Armstrong E.J., Feldman D.N., Wang T.Y., Kaltenbach L.A., Yeo K.-K., Wong S.C., Spertus J., Shaw R.E., Minutello R.M., Moussa I. (2012). Clinical Presentation, Management, and Outcomes of Angiographically Documented Early, Late, and Very Late Stent Thrombosis. JACC Cardiovasc. Interv..

[63] Katsikis A., Keeble T.R., Davies J.R., Jagathesan R., Kabir A., Sayer J.W., Robinson N.M., Kalogeropoulos A.S., Aggarwal R.K., Gamma R.A. (2020). Contemporary management of stent thrombosis: Predictors of mortality and the role of new-generation drug-eluting stents. Catheter. Cardiovasc. Interv..

[64] Neumann F.J., Sousa-Uva M., Ahlsson A., Alfonso F., Banning A.P., Benedetto U., Byrne R.A., Collet J.P., Falk V., Head S.J. (2019). 2018 ESC/EACTS Guidelines on myocardial revascularization. Eur. Heart J..

[65] Lawton J.S., Tamis-Holland J.E., Bangalore S., Bates E.R., Beckie T.M., Bischoff J.M., Bittl J.A., Cohen M.G., DiMaio J.M., Don C.W. (2022). 2021 ACC/AHA/SCAI Guideline for Coronary Artery Revascularization: Executive Summary: A Report of the American College of Cardiology/American Heart Association Joint Committee on Clinical Practice Guidelines. Circulation.

[66] Alfonso F., Dutary J., Paulo M., Gonzalo N., Pérez-Vizcayno M.J., Jiménez-Quevedo P., Escaned J., Bañuelos C., Hernández R., Macaya C. (2012). Combined use of optical coherence tomography and intravascular ultrasound imaging in patients undergoing coronary interventions for stent thrombosis. Heart.

[67] Souteyrand G., Amabile N., Mangin L., Chabin X., Meneveau N., Cayla G., Vanzetto G., Barnay P., Trouillet C., Rioufol G. (2016). Mechanisms of stent thrombosis analysed by optical coherence tomography: Insights from the national PESTO French registry. Eur. Heart J..

[68] Gori T., Polimeni A., Indolfi C., Räber L., Adriaenssens T., Münzel T. (2019). Predictors of stent thrombosis and their implications for clinical practice. Nat. Rev. Cardiol..

[69] Lee C.W., Kang S.-J., Park D.-W., Lee S.-H., Kim Y.-H., Kim J.-J., Park S.-W., Mintz G.S., Park S.-J. (2010). Intravascular Ultrasound Findings in Patients with Very Late Stent Thrombosis After Either Drug-Eluting or Bare-Metal Stent Implantation. J. Am. Coll. Cardiol..

[70] Taniwaki M., Radu M.D., Zaugg S., Amabile N., Garcia-Garcia H.M., Yamaji K., Jørgensen E., Kelbæk H., Pilgrim T., Caussin C. (2016). Mechanisms of Very Late Drug-Eluting Stent Thrombosis Assessed by Optical Coherence Tomography. Circulation.

[71] Adriaenssens T., Joner M., Godschalk T.C., Malik N., Alfonso F., Xhepa E., De Cock D., Komukai K., Tada T., Cuesta J. (2017). Optical Coherence Tomography Findings in Patients with Coronary Stent Thrombosis: A Report of the PRESTIGE Consortium (Prevention of Late Stent Thrombosis by an Interdisciplinary Global European Effort). Circulation.

[72] Jia H., Dai J., He L., Xu Y., Shi Y., Zhao L., Sun Z., Liu Y., Weng Z., Feng X. (2022). EROSION III: A Multicenter RCT of OCT-Guided Reperfusion in STEMI With Early Infarct Artery Patency. JACC Cardiovasc. Interv..

[73] Jia H., Dai J., Hou J., Xing L., Ma L., Liu H., Xu M., Yao Y., Hu S., Yamamoto E. (2017). Effective anti-thrombotic therapy without stenting: Intravascular optical coherence tomography-based management in plaque erosion (the EROSION study). Eur. Heart J..

[74] Xing L., Yamamoto E., Sugiyama T., Jia H., Ma L., Hu S., Wang C., Zhu Y., Li L., Xu M. (2017). EROSION Study (Effective Anti-Thrombotic Therapy Without Stenting: Intravascular Optical Coherence Tomography-Based Management in Plaque Erosion): A 1-Year Follow-Up Report. Circ. Cardiovasc. Interv..

[75] He L., Qin Y., Xu Y., Hu S., Wang Y., Zeng M., Feng X., Liu Q., Syed I., Demuyakor A. (2021). Predictors of non-stenting strategy for acute coronary syndrome caused by plaque erosion: Four-year outcomes of the EROSION study. EuroIntervention.

[76] Hu S., Wang C., Zhe C., Zhu Y., Yonetsu T., Jia H., Hou J., Zhang S., Jang I.-K., Yu B. (2017). Plaque erosion delays vascular healing after drug eluting stent implantation in patients with acute coronary syndrome: An In Vivo Optical Coherence Tomography Study. Catheter. Cardiovasc. Interv..

[77] Fujino A., Mintz G.S., Matsumura M., Lee T., Kim S.-Y., Hoshino M., Usui E., Yonetsu T., Haag E.S., Shlofmitz R.A. (2018). A new optical coherence tomography-based calcium scoring system to predict stent underexpansion. EuroIntervention.

[78] Guedeney P., Claessen B.E., Mehran R., Mintz G.S., Liu M., Sorrentino S., Giustino G., Farhan S., Leon M.B., Serruys P.W. (2020). Coronary Calcification and Long-Term Outcomes According to Drug-Eluting Stent Generation. JACC Cardiovasc. Interv..

